# Psychometric Properties of Measuring Antiretroviral Therapy Adherence Among Young Latino Sexual Minority Men With HIV: Ecological Momentary Assessment and Electronic Pill Dispenser Study

**DOI:** 10.2196/51424

**Published:** 2024-11-21

**Authors:** Diana M Sheehan, Tendai Gwanzura, Cynthia Ibarra, Daisy Ramirez-Ortiz, Dallas Swendeman, Dustin T Duncan, Miguel Muñoz-Laboy, Jessy G Devieux, Mary Jo Trepka

**Affiliations:** 1 Department of Epidemiology, Florida International University, 11200 SW 8th Street, Miami, FL, 33199, United States, 1 3053488068; 2 Research Centers in Minority Institutions, Florida International University, Miami, FL, United States; 3 Semel Institute for Neuroscience and Human Behavior, University of California, Los Angeles, CA, United States; 4 Department of Epidemiology, Mailman School of Public Health, Columbia University, New York City, NY, United States; 5 School of Social Welfare, Stony Brook, Stony Brook, NY, United States; 6 Department of Health Promotion and Disease Prevention, Florida International University, Miami, FL, United States

**Keywords:** human immunodeficiency virus, HIV, MSM, sexual minority, antiretroviral therapy, Latino, Hispanic, adherence, psychometric, ecological momentary assessment, electronic pill dispenser, validity, acceptability, compliance, medication dispenser, reminder, alert, digital health, young adult

## Abstract

**Background:**

Increasing HIV rates among young Latino sexual minority men (YLSMM) warrant innovative and rigorous studies to assess prevention and treatment strategies. Ecological momentary assessments (EMAs) and electronic pill dispensers (EPDs) have been used to measure antiretroviral therapy (ART) adherence repeatedly in real time and in participants’ natural environments, but their psychometric properties among YLSMM are unknown.

**Objective:**

The study’s objective was to assess the concurrent validity, acceptability, compliance, and behavioral reactivity of EMAs and EPDs among YLSMM with HIV.

**Methods:**

A convenience sample of 56 YLSMM with HIV with suboptimal ART adherence, aged 18‐34 years, was recruited into a 28-consecutive-day EMA study. Concurrent validity was analyzed by comparing median ART adherence rates and calculating Spearman correlations between ART adherence measured by EMA, EPD, and baseline retrospective validated 3-item and single-item measures. Acceptability was assessed in exit interviews asking participants to rate EMA and EPD burden. Compliance was assessed by computing the percent lost to follow-up, the percent of EMAs missed, and the percentage of days the EPD was not opened that had corresponding EMA data self-reporting adherence to ARTs. Behavioral reactivity was assessed by computing the median change in ART adherence during the study period, using generalized mixed models to assess whether the cumulative number of EMAs completed and days of EPD use predicted ART adherence over time, and by asking participants to rate perceived reactivity using a Likert scale.

**Results:**

EMA ART adherence was significantly correlated with baseline validated 3-item (*r*=0.41, *P*=.003) and single-item (*r*=0.52, *P*<.001) measures, but correlations were only significant for participants that reported EMA was not burdensome. Correlations for EPD ART adherence were weaker but significant (*r*=0.36, *P*=.009; *r*=0.34, *P*=.01, respectively). Acceptability was high for EMAs (48/54, 89%) and EPDs (52/54, 96%) per self-report. Loss to follow-up was 4% (2/56), with the remaining participants completing 88.6% (1339/1512) of study-prompted EMAs. The percentage of missed EMA surveys increased from 5.8% (22/378) in week 1 of the study to 16.7% (63/378) in week 4. Of 260 days when EPDs were not opened, 68.8% (179) had a corresponding EMA survey self-reporting ART adherence. Reactivity inferred from the median change in ART adherence over time was 8.8% for EMAs and −0.8% for EPDs. Each completed EMA was associated with 1.03 odds (95% CI 1‐1.07) of EMA ART adherence over time, and each day of EPD use with 0.97 odds (95% CI 0.96‐0.99) of EPD ART adherence over time. Self-reported perceived behavioral reactivity was 39% for EMAs and 35% for EPDs.

**Conclusions:**

This study provides evidence of concurrent validity with retrospective validated measures for EMA- and EPD-measured ART adherence among YLSMM, when participant burden is carefully considered, without significant behavioral reactivity. While acceptability and compliance of EMAs and EPDs were high overall, noncompliance increased over time, suggesting respondent fatigue.

## Introduction

In 2020, Latinos accounted for 27% of new HIV diagnoses in the United States and 31% of new HIV diagnoses among sexual minority men (SMM) [1]. In the same year, Latino SMM accounted for 23% (n=246,097) of people with HIV in the United States [1]. Despite viral suppression being critical to prevent new cases of HIV and preserve the health of people with HIV [2], only 66% of Latino SMM with HIV in the United States achieved viral suppression in 2020, compared with 73% among non-Latino White SMM [3]. Low rates of viral suppression are partially due to suboptimal antiretroviral therapy (ART) adherence. The US representative sample data from 2015 to 2019 showed only 57% of Latino SMM with diagnosed HIV reported taking all their ART doses in the previous month, with younger (<40 years old) Latino SMM less likely to report being adherent [4].

Ecological momentary assessments (EMAs) collect data on behaviors and experiences repeatedly in real time and in the participant’s natural environment [5]. Electronic pill dispensers (EPDs) are pillboxes designed to monitor pill-taking by recording timestamped signals when the device is opened [6]. EPDs have been used for nearly 2 decades as an objective measure of ART adherence in rigorous HIV and pre-exposure prophylaxis clinical trials that have had significant implications [7-10]. While the validity, acceptability, and compliance of EMA and EPD have been evaluated in some studies suggesting they are valid and acceptable tools to measure outcomes among the general population of people with HIV [11-15], the psychometric properties of EMA and EPD among young (18‐34 years old) SMM are unknown. Acceptability and compliance of these demanding protocols may be lower for those experiencing socioeconomic and structural barriers [13] such as young SMM, which may compromise the validity of the data collected. A 2019 study of young SMM and trans women in San Francisco showed those reporting housing instability, foregoing HIV medications to afford basic needs, or reporting lower educational levels were more likely to miss EMA surveys [13].

Both EMA- and EPD-measured ART adherence pose the additional psychometric concern of reactivity, or the potential effect that repeated and frequent measurements of adherence behavior will have on the observed outcome despite a lack of intervention [16]. Behavioral reactivity has been examined more extensively in EMA studies of drinking and smoking, which have shown no or small reactivity effects, that is, none or a small decrease in drinking or smoking over the course of the EMA study assumed to be due to repeated real-time monitoring of the behavior [17-20]. Studies of behavioral reactivity from the use of EPD to monitor medication adherence across various health conditions have also reported no or minimal reactivity when used without additional intervention components, suggesting minimal concern about its use in observational research [21,22]. However, we were unable to identify any studies assessing the behavioral reactivity of EMAs or EPDs when used to measure ART adherence among Latino SMM, suggesting an important gap in the literature.

In this study, we report findings of 3 study objectives that assess the rigor and use of EMA and EPD and inform methodological and analytical choices in the study of ART adherence among young (18‐34 years old) Latino SMM (YLSMM). Our first objective was to assess the concurrent validity of EMA- and EPD-measured ART adherence by comparing their agreement with retrospective self-reported validated and single-item measures of ART adherence taken at baseline. Based on previous research, we hypothesized that EMA and EPD ART adherence would be significantly correlated with validated baseline ART adherence measures [23,24]. Our second objective was to assess acceptability and compliance with EMA and EPD ART adherence protocols among this population. We hypothesized that acceptability and compliance with daily EMAs and EPD use would be moderate, given previous research showing high acceptance, but cautioned by potentially added barriers experienced by our population [13,25]. Our final objective was to assess behavioral reactivity to daily ART adherence monitoring. We hypothesized improvements in ART adherence over the course of the study and that participants would self-report a change in ART adherence behaviors due to their participation in the EMA and EPD protocols [21,22].

## Methods

### Study Design and Procedures

We conducted an EMA study of YLSMM with HIV to examine ART adherence behaviors and barriers to daily ART adherence [26]. After consent, participants completed an online self-administered baseline questionnaire, followed by 28 days of EMAs, and a telephone mixed methods (qualitative and quantitative) exit interview. EMAs consisted of 25 questions that took participants 4‐6 minutes to complete daily and were delivered via a link in an SMS text message at 5 PM with 2 subsequent reminders 30 minutes apart. We chose to deliver the EMAs at 5 PM for all participants to ensure that the survey questions were answered in a similar way, with the same relative time point as a reference, in mind. For example, when answering questions about their mood, they were all referring primarily to their mood that day, versus the previous day if the EMA was delivered in the early morning for some. Study staff monitored incoming EMAs daily, and participants who did not complete 3 or more consecutive surveys were contacted via phone to remind them or troubleshoot issues. Similar to other studies, during enrolment, participants were offered an additional incentive if at least 80% of surveys were completed [27,28]. Baseline, EMA, and exit surveys were completed in the participant’s preferred language (English or Spanish). When available, validated translations were used. If not available, questions were translated by a native Spanish speaker team member and reviewed by 2 additional Spanish speakers from different countries of origin, and differences were discussed until a consensus was reached. In addition, participants were given a Wisepill 3G RT2000 Dispenser with a 7-compartment cartridge (fitting at least 7 pills at a time per 1 week of ARTs) and a charger and asked to use the EPD daily to store their ARTs throughout the 28-day protocol. EPDs recorded date, time, and geolocation when opened. EPDs were monitored daily by study staff for battery power and signal; if a signal was not detected, study staff contacted participants via phone to troubleshoot or exchange the EPD, if needed.

### Recruitment

A convenience sample of 56 YLSMM was recruited from the South Florida area between February 2021 and May 2022. The sample size was calculated to provide 80% power to detect meaningful odds ratios for the association between time-varying discrete covariates in the primary aims of the parent study and ART adherence as a binary outcome. Participants were recruited via referrals from case managers, linkage specialists, and physicians at 3 community HIV clinics, by posting flyers at local clinics and social service agencies serving people with HIV, and through paid targeted online advertising on search engines (eg, Google) and social media platforms (eg, Facebook). Eligible participants included individuals aged 18‐34 years who self-identified as Latino; who self-identified as gay, bisexual, or other SMM; who spoke either English or Spanish; whose HIV was confirmed through a case manager referral, by submission of an HIV laboratory test, or ART prescription by the participant; who had a current ART prescription; who had no AIDS diagnosis; and who had no activity restrictions. Additionally, eligible participants needed to meet one of two criteria for suboptimal ART adherence: evidence of at least one detectable viral load test (≥20 copies/mL) in the past 24 months, or self-report ART adherence of less than “excellent” or “very good” in a 6-point Likert rating scale.

### Measures

We measured adherence in 4 ways. First, we calculated *EMA ART adherence* using responses to the following question on daily EMAs—“Since the last survey, have you missed a dose of your HIV medication?” (yes/no)—and defined it as the total number of days that ARTs were taken divided by the total number of days a survey was submitted, setting missed surveys (173/1512) to missing. Second, we calculated *EPD ART adherence* as the total number of days the EPD was opened divided by the total number of days the EPD was on and receiving a signal, setting days when the EPDs were out of signal range (11/1512) to missing [7]. Third, we calculated *validated 3-item self-reported retrospective ART adherence* at baseline using Wilson et al’s [29] ART adherence scale. Extensive research has been conducted to validate self-reported ART adherence by assessing its relationship with HIV viral load, suggesting self-report is a valid way to measure ART adherence [8,14,23,29,30]. Wilson et al’s [29] scale has been shown to have a positive linear association with viral suppression in clinical and research settings. The three questions in the scale are as follows. (1) “In the past 30 days, on how many days did you miss at least one dose of any of your HIV medicines?” (2) “In the past 30 days, how good a job did you do at taking your HIV medicines in the way you were supposed to?” (6-point Likert from “very poor” to “excellent”). (3) “In the past 30 days, how often did you take your HIV medicines in the way you were supposed to?” (6-point Likert scale from “always” to “never”). We coded each question in Wilson et al’s [29] scale so that higher numbers corresponded to greater adherence, then transformed responses to a 0‐100 scale and took the mean of the 3 transformed items. Finally, we calculated *single-item retrospective ART adherence* using only question one of the Wilson et al [29] scale because it was adapted to be used in the EMAs. Median percent adherent was used for all measures due to the skewness of the data.

Measures of acceptability were gathered from the exit interview by asking participants to state their level of agreement (5-point Likert scale from “strongly agree” to “strongly disagree”) with the following 2 statements: “It was burdensome to complete the surveys daily” and “It was burdensome to use the electronic pillbox daily.” Self-reported measures of behavioral reactivity were also obtained from the exit interview by asking participants to state their level of agreement (5-point Likert scale from “strongly agree” to “strongly disagree”) to the following 2 statements: “The daily surveys affected my HIV medication adherence” and “The use of the electronic pillbox affected my HIV medication adherence.” We categorized acceptability and reactivity measures as “yes” burdensome or “yes” affected adherence if they responded, “strongly agree” or “agree.”

### Data Analysis

We assessed concurrent validity, acceptability, compliance, and behavioral reactivity of EMAs and EPDs among YLSMM with HIV. *Concurrent validity* was assessed by comparing median adherence rates and computing Spearman correlations for the whole sample and partial correlations by age, race, education, and US-born status between EMA-EPD-ART adherence and both the validated 3-item and single-item retrospective ART adherence measures [29]. We also stratified our correlation analysis by reporting the burden of EMAs and EPDs to capture if validity was impacted by the acceptability of EMA and EPD protocols. The tetrachoric correlation between EMAs and EPDs was calculated to assess the day-to-day agreement between the EMAs and EPDs across all participants and all 1512 observations. Correlations were considered significant if the *P* value was <.05 [8]. We conducted sensitivity analyses to understand the impact of missing EMA and EPD data on validity by replacing missing EMA adherence data with EPD data for the same day, if available. Similarly, we assessed the impact of replacing days without an “open” signal in the EPD (originally coded as nonadherent days) with EMA data for the same day, if available.

*Acceptability* was analyzed as the proportion of participants who reported the EMA and EPD to be burdensome. To assess *compliance*, we computed the percent of missed EMA surveys for the 28-day period and by study week, the percent of days the EPD was not opened that had corresponding EMA data self-reporting adherence to ARTs, and the percent lost to follow-up. Finally, *behavioral reactivity* was assessed objectively by computing the median change in EMA- and EPD-measured ART adherence over the 28-day period and by using generalized mixed models to assess whether the cumulative number of EMAs completed predicted ART adherence over time. Behavioral reactivity was also assessed subjectively as the proportion of participants who self-reported that the use of the EMA and EPD affected their ART adherence behavior.

### Ethical Considerations

The Florida International University institutional review board approved this study (IRB-18-0296). Informed consent was obtained from all participants in their preferred language (English/Spanish) prior to their participation in this study. Measures to protect the privacy and confidentiality of the data and participants included removing personally identifiable information from all data collection instruments, encrypting data during transfer and storage, storing data on secure systems with restricted access, and limiting access to identifiable information to the study coordinator and principal investigator. Participants were compensated US $35 for completing the baseline questionnaire, US $150 for participating in the 28-day EMA, US $35 for completing the exit interview, and US $30 if they completed at least 80% of EMA surveys for a maximum total of US $250.

## Results

### Participants

Of 56 participants who consented to be part of the study and completed the baseline questionnaire, 54 began the EMA and EPD portion of the study and were considered in the current analyses (details provided in the *Acceptability and Compliance* section). Twenty-four participants completed the EMAs in Spanish. Participants (n=54) had a mean age of 28.8 (SD 3.4) years and predominantly reported their gender identity to be male (52/54, 96%) and sexual identity to be gay (47/54, 87%) (Table 1). Participant’s Latino origin was predominantly from South America (22/54, 41%) and Cuba (16/54, 30%), most self-identified as White (30/54, 56%) or multiracial or other (20/54, 37%), and most were foreign-born (37/54, 69%). About 44% (24/54) were single, 37% (20/54) had a college degree, and 50% (27/54) were working full-time.

**Table 1.** Characteristics of young Latino sexual minority men with HIV who participated in a 28-day EMA^a^ and EPD^b^ ART^c^ adherence monitoring study (N=54^d^).

Characteristics		Values
Age (year), mean (SD)		28.8 (3.4)
Gender identity, n (%)		
	Male	52 (96)
	Other	2 (4)
Sexual identity, n (%)		
	Gay	47 (87)
	Bisexual	2 (4)
	Other	5 (9)
Latino origin, n (%)		
	Cuban	16 (31)
	South American	22 (42)
	Central American	8 (15)
	Other or mixed	6 (12)
Race, n (%)		
	White	30 (56)
	Black	4 (7)
	Other or multiracial	20 (37)
Immigrant generation, n (%)		
	First generation (foreign-born)	37 (71)
	Second or third generation (US-born)	14 (27)
	Don’t know or prefer not to respond	1 (2)
Year of stay in the United States (if foreign-born), n (%)		
	1‐5 years	22 (41)
	6‐10 years	13 (24)
	>10 years	19 (35)
Marital status, n (%)		
	Same-sex partner, married	11 (20)
	Same-sex partner, unmarried	19 (35)
	Single	24 (44)
Education, n (%)		
	High school diploma or less^e^	9 (17)
	Some college or vocational school	17 (31)
	Bachelor’s degree	20 (37)
	Master’s or doctoral degree	4 (7)
	Unknown or prefer not to respond	4 (7)
Employment status, n (%)		
	Full-time	27 (50)
	Part-time	11 (20)
	Self-employed	4 (7)
	Student	3 (6)
	Unemployed	8 (15)
	Don’t know or prefer not to respond	1 (2)

a EMA: ecological momentary assessment.

b EPD: electronic pill dispenser.

c ART: antiretroviral therapy.

d Two participants were excluded because they did not start the EMA or EPD protocol.

e One participant reported completing grades 1‐11 but no high school diploma.

### Validity

Of the 54 participants who started the EMA and EPD protocol, 52 had reliable data to be used in the assessment of validity (details provided in the *Acceptability and Compliance* section). All 52 participants reported being on once-daily ART regimens. Median EMA ART adherence was highest (100%, IQR 95.2%-100%), followed by single-item retrospective ART adherence (96.7%, IQR 93.3%-100%) (Table 2). Median adherence was lowest and nearly identical between the validated 3-item retrospective measure (89.2%, IQR 82.9%-95.4%) and the EPD (89.3%, IQR 75%-96.4%). EMA ART adherence was significantly correlated with the validated 3-item retrospective ART adherence measure (0.41, *P*=.003), the single-item retrospective ART adherence measure (0.52, *P*<.001), and the EPD measure (0.45, *P*<.001). The correlations between EPD ART adherence and both baseline ART adherence measures were weaker but also significant (validated 3-item measure=0.36, *P*=.009; single-item measure=0.34, *P*=.01). Partial correlations controlling for age, race, education, and US-born status were not appreciably different (not shown in the table). The tetrachoric correlation coefficient between EMA ART adherence and EPD ART adherence for all observations (n=1306) across the whole sample (n=52) was 0.41 (*P*=.003).

In sensitivity analyses, when we imputed missing EMA ART adherence data with EPD data, the correlation between EMA ART adherence and validated 3-item ART adherence (0.46, *P*<.001) and single-item ART adherence (0.54, *P*<.001) remained nearly the same. When we imputed data for days where the EPD was not opened with EMA data, the correlation between EPD ART adherence and validated 3-item ART adherence (0.35, *P*=.01) remained the same, but the correlation with the single-item ART adherence measure improved from 0.34 (*P*=.01) to 0.42 (*P*=.002).

To assess whether validity was a function of EMA or EPD acceptability, we assessed validity by the reported burden of EMA and EPD use. The Spearman correlation coefficient between EMA ART adherence and validated 3-item retrospective ART adherence was significant and positive (0.49, *P*<.001) among those who reported participating in daily EMAs was not burdensome compared to not significant for those who reported the EMAs were burdensome (−0.71, *P*=.12). A similar pattern was found when comparing EMA ART adherence with the single-item retrospective ART adherence measure by reported burden (not burdensome: 0.55, *P*<.001; burdensome: 0.09, *P*=.86). Among those who found the EPD not to be burdensome, the Spearman correlations between EPD ART adherence and validated 3-item retrospective ART adherence and single-item retrospective ART adherence were nearly unchanged compared to the overall sample. We were unable to assess the validity of EPD for those who found the EPD to be burdensome due to the small number of participants who reported the EPD to be burdensome (n=2).

**Table 2.** Validity of EMA^a^ and EPD^b^ ART^c^ adherence among young Latino sexual minority men with HIV.

	Total, n	Median percent ART adherence^d^ (IQR)	Spearman correlation coefficients (*P* value)		
			Validated 3-item retrospective ART adherence	Single-item retrospective ART adherence	EPD ART adherence
Daily prospective measures					
EMA ART adherence	52^e^	100.00 (95.24-100)	0.41 (.003)	0.52 (<.001)	0.45 (<.001)^f^
Not burdensome	49	—^g^	0.49 (<.001)	0.55 (<.001)	—
Burdensome	6	—	−0.71 (.12)	0.09 (.86)	—
EPD ART adherence	52^e^	89.29 (75.00-96.43)	0.36 (.009)	0.34 (.01)	—
Not burdensome	53	—	0.37 (.009)	0.35 (.01)	—
Burdensome	2	—	N/A^h^	N/A	—
Baseline retrospective measures					
Validated 3-item ART adherence scale	—	89.17 (82.92-95.42)	—	0.76 (<.001)	—
Single-item ART adherence	—	96.67 (93.34-100)	—	—	—

a EMA: ecological momentary assessment.

b EPD: electronic pill dispenser.

c ART: antiretroviral therapy.

d Median of within-person 28-day percent adherence.

e Two participants were excluded for not initiating the EMA and EPD protocol, 1 participant was excluded because of missing data on 23 of 28 EMA days, and another 1 participant was excluded for providing identical responses on 22 of 28 EMA days.

f Tetrachoric correlation coefficient between daily EMA ART adherence responses (dichotomized, yes=1, no=0) and daily EPD recorded adherence (dichotomized, yes=1, no=0) for all observations (n=1306) across the whole sample (n=52) was 0.41 (*P*=.003).

g Not applicable.

h N/A: not available; only 2 participants reported the electronic pill dispenser to be burdensome, thus correlations coefficients were not able to be computed.

### Acceptability and Compliance

Approximately 89% (48/54) of participants self-reported that the daily EMA surveys were not burdensome, 96% (52/54) reported that using the EPD was not burdensome, and 98% (53/54) that they would participate in a similar study in the future (Table 3). Participants were highly compliant with the EMA protocol. Among 56 participants, 2 were lost to follow-up prior to starting the EMA or EPD protocol. The remaining 54 participants responded to 1339 (88.6%) of 1512 study-prompted EMA surveys over the 28-day study protocol. The EMA nonresponse rate ranged from 0 to 23 days, with a median number of days missed of 2. The number of participants who did not respond to the EMA survey increased from 1 participant on the first EMA day to 10 participants on day 28, with some inconsistency in days 16 and 19. The percentage of missed EMA surveys increased from 5.8% (22/378) in week 1, to 10.6% (40/378) in week 2, 12.2% (46/378) in week 3, and 16.7% (63/378) in week 4. Regarding data quality, 1 participant had missing data on 23 EMA days, and a second participant had identical responses on 22 of 28 EMA days and the responses were inconsistent across questions within the same survey. EPD devices recorded a signal (ie, they were on, charged, and recording data) for 1501 (99.3%) of 1512 days of data collection across all participants. EPD devices were recording data but not opened on a total of 260 (17.3%) of 1501 days. Among days when the EPDs were not opened, 179 (68.8%) had EMA data self-reporting adhering to ART, 23 (8.8%) had EMA data reporting not adhering to ART, and 58 (22.3%) had missing EMA data. Moreover, the proportion of days when EPDs were not opened but ART adherence was reported on EMA surveys increased steadily from 80% in week 1, to 89.7% in week 2, 91.1% in week 3, and 92.7% in week 4. In post hoc analyses, to assess potential changes in the use of the EPD, we examined the tetrachoric correlation between EMA ART adherence and EPD ART adherence by study week. The correlation was highest in week 1 (0.54) and lowest in week 4 (0.34) but fluctuated in the middle weeks (week 2=0.36; week 3=0.42).

**Table 3.** Self-reported acceptability and behavioral reactivity of EMA^a^ and EPD^b^ ART^c^ adherence monitoring among young Latino sexual minority men with HIV (N=54^d^).

Statement	Participants, n (%)
Acceptability	
“It was burdensome to complete the surveys daily”	
Yes^e^	6 (11)
No^f^	48 (89)
“It was burdensome to use the electronic pillbox daily”	
Yes	2 (4)
No	52 (96)
“I would participate in a similar study again”	
Yes	53 (98)
No	1 (2)
Reactivity	
“The daily surveys affected my HIV medication adherence”	
Yes	21 (39)
No	33 (61)
“The use of the electronic pillbox affected my HIV medication adherence”	
Yes	19 (35)
No	35 (65)
“I changed the way I take my medication as a result of participating in this study”	
Yes	40 (74)
No	14 (26)

a EMA: ecological momentary assessment.

b EPD: electronic pill dispenser.

c ART: antiretroviral therapy.

d Two participants were excluded because they did not start the EMA or EPD protocol. The participant who did not complete 23 of 28 EMAs and another participant who had identical responses on 22 of 28 EMA surveys were included in the analysis of acceptability and compliance.

e Yes: “strongly agree” and “agree.”

f No: “neither agree nor disagree,” “disagree,” and “strongly disagree.”

### Behavioral Reactivity

The median change between baseline (validated 3-item retrospective ART adherence) and end-of-study EMA adherence (difference between the measures) was 8.75% (IQR 1.36%-16.14%); using single-item retrospective ART adherence, the median change was 0% (IQR -1.99%-1.99%). The median change between baseline and end-of-study EPD adherence was −0.8% (IQR -9.7%-8.1%) using the validated 3-item measure and −7.14% (IQR -16.54%-2.26%) using the single-item measure. When we assessed whether the number of surveys completed predicted EMA ART adherence in a generalized mixed model, each increasing day of EMAs completed was associated with a 1.03 (95% CI 1‐1.07) increased odds of ART adherence over time. Controlling for baseline adherence (3-item scale) did not change this association. Each increasing day of EPD use was associated with 0.97 (95% CI 0.96‐0.99) decreasing odds of EPD ART adherence over time, without change when controlling for baseline adherence. Self-reported reactivity is reported in Table 3. Approximately, 39% (21/54) of participants self-reported that their ART adherence changed over the course of the study due to the EMAs, and 35% (19/54) due to their use of the EPD. The relationship between EMA surveys submitted or days of EPD use and ART adherence was not different by self-reported reactivity level.

## Discussion

### Principal Findings

This comprehensive study had several primary findings in 3 key methodological areas: validity, acceptability and compliance, and behavioral reactivity. This study provided evidence of concurrent validity of baseline retrospective validated ART adherence measures for EMA- and EPD-measured ART adherence among YLSMM. The strength of the correlation between EMA-measured ART adherence and our baseline measures was lower than the relatively high correlation (0.7) found in a study using a 14-day EMA protocol among adults with HIV [11]. Our study may have observed a lower correlation because of the additional burden of completing 28 days of EMA, as we only found concurrent validity to be significant among participants who reported the EMA protocol was not burdensome. However, the strength of the correlation between EPD-measured ART adherence and baseline measures in our study was similar to 2 other studies comparing EPD-measured ART adherence to baseline measures, pharmacy records, and viral load [8,14]. Validity did not appreciably improve when combining EMA and EPD data (imputing missing values across these 2 data collection modalities), except for a small increase in the correlation between EPD-measured ART adherence and the single-item retrospective measure, suggesting a potential benefit of using EMA data to impute missing EPD values.

Self-reported acceptability of both the EMA and EPD was high among YLSMM in this study. While compliance overall reached the suggested 80% target [31] and mirrored or surpassed the high compliance in previous studies [31,32], noncompliance with EMA surveys nearly tripled in a 4-week period suggesting respondent fatigue. Interestingly, a meta-analysis by Jones et al [31] of EMA studies among substance users did not find evidence that EMA compliance was associated with the daily frequency of EMAs or length of assessments. However, the structure of financial incentives may influence compliance. Our study offered an additional incentive at the end of the study if at least 80% of EMAs were completed which may have helped to reach overall high compliance, but other incentive structures used in previous research, such as weekly disbursements of payments, or payments associated with each completed survey [33], may have helped to maintain high compliance across time. It is worth noting the discrepancy between high self-reported acceptability of EMA expressed during the exit interviews and decreasing EMA compliance over time, as this inconsistency was also found in a study of YLSMM and trans women in San Francisco [13]. Our finding that participants self-reported ART adherence in EMA surveys on nearly 70% of days that EPDs were not opened, that this discrepancy increased over time, and that concurrent validity of EPD-measured ART adherence improved when supplemented with self-reported EMA data, suggest that some participants were not using the EPD every day. It is possible that even though participants did not perceive the EMA or EPD as “burdensome,” they still forgot or chose not to complete the EMA surveys on some days and not use the EPD. In an earlier qualitative phase of this study [26] used, in part, to obtain feedback on the EMA or EPD protocols, some participants expressed concerns about the size of the EPD, the feasibility of carrying it with them, and the potential for disclosing their HIV status if it was seen.

Similar to previous research, our study did not find evidence of behavioral reactivity from the use of EMA, as ART adherence did not increase with the increasing number of completed EMA surveys [21,22]. While we found an association between the increasing number of days of EPD use and decreases in ART adherence over time, the effect was small and only marginally significant (odds ratio 0.97, 95% CI 0.96‐0.99). This seemingly paradoxical association provides additional evidence of decreases in the use of EPD over time and is consistent with our EPD compliance data that suggests participants are in fact adhering to their ARTs even on days when they do not open the EPD. For example, participants may remove more than 1 pill at a time from the EPD for later dosing and thus not open the EPD daily, a behavior referred to as “pocket dosing” [34,35]. Although about a third of participants self-reported that their ART adherence changed because of the use of EMA or EPD, we did not find that behavioral reactivity measured objectively was stronger for those that self-reported behavioral reactivity, suggesting self-reflection of ART adherence changes may not necessarily concur with actual ART adherence changes measured via EMA or EPD.

### Limitations

Our study findings have some limitations. For our assessment of validity, we compared EMA or EPD ART adherence to baseline past 30-day ART adherence; thus, the time periods of measurement did not overlap. This limitation would have been especially problematic if we had found significant behavioral reactivity from the use of the EMA or EPD, but we did not find this to be the case in our sample. Further, our study did not ask participants to report ART doses taken outside while not using the EPD, a limitation reflected in the high proportion of days when the EPD was not opened but a dose was reported as taken in the same day’s EMA survey. Additionally, as indicated by 1 participant with the same responses for all survey questions across 23 EMA surveys, asking the same questions each day for 28 days may fatigue participants, and although EMA compliance was high, the quality of the responses may have decreased over time; this limitation would be especially problematic when measuring factors expected to vary daily such as mental health or substance use indicators. Future studies may want to consider randomizing the order of questions daily or creating a variable schedule of questions with a larger sample of YLSMM. It is worth noting that our participants started the study on varying days of the week and compliance with EMA or EPD may differ by day of the week (weekdays vs weekends). Finally, the convenience sample recruited at clinics and online limits the generalizability of the findings, and the very high ART adherence and few missed doses in the sample may have limited power in our analyses, particularly in our evaluation of behavioral reactivity.

### Implications for Research

Our findings highlight the importance of developing EMA protocols that are not burdensome for participants to ensure the validity of ART adherence measurements and compliance with protocols over time. Our findings also imply the need to carefully track compliance of EMA or EPD protocols over time objectively, and not only with perceived acceptability measures, and the need to consider decreases in compliance in the analysis of findings. As suggested by others, the choice of an EPD may enhance or diminish compliance, and study and participant characteristics should be considered when selecting an appropriate device for the target population [36]. Additionally, our findings imply that measuring ART adherence via EMAs or EPDs among YLSMM is not associated with unintentional changes in behaviors in observational studies and that these protocols on their own may not be sufficient to obtain ART adherence improvements in experimental settings. Ecological momentary interventions seeking to increase ART adherence among YLSMM should consider building additional intervention components on top of a simple 1-way text message question about ART adherence behavior. Two-way text message ART adherence reminders have shown promise with youth and may be worth testing among YLSMM [37], as well as interventions that address other adherence barriers such as unmet basic needs, substance use, and mental health conditions [38]. Combined, these interventions can address the primary reasons for missed ART doses among YLSMM including forgetting to take medication, a change in routine, oversleeping, drug use, depression, and lack of needed ancillary services [4,26]. The low cost and easy implementation of self-reported measures of medication adherence using mobile health, such as the one used in our EMA, make it a potentially critical tool for large-scale studies, clinical settings, or for scaling interventions to the community [6,39,40]. Conversely, medication adherence monitoring technologies can be costly, making their application and broad applicability to clinical and community settings limited [6]. In choosing a method for adherence monitoring, EMAs may be better suited for short-term monitoring of larger populations, or in studies where the target population is likely to find EPD challenging (eg, people who are homeless, or in communities with high levels of HIV stigma). EPDs may be more useful for long-term monitoring of adherence in smaller samples.

### Conclusions

In conclusion, this study provides evidence of concurrent validity with retrospective validated measures for EMA- and EPD-measured ART adherence among YLSMM, when participant burden is carefully considered, without significant behavioral reactivity. While acceptability and compliance of EMAs and EPDs were high overall, noncompliance increased over time, suggesting respondent fatigue. To ensure rigor and data quality, compliance with EMA and EPD protocols should be carefully tracked, incentivized, and incorporated into the data analysis plan.

## Abbreviations

ART: antiretroviral therapy
EMA: ecological momentary assessment
EPD: electronic pill dispenser
SMM: sexual minority men
YLSMM: young Latino sexual minority men
